# The Impact of Ophthalmic Lens Power and Treatments on Eye Tracking Performance

**DOI:** 10.3390/jemr19010004

**Published:** 2025-12-29

**Authors:** Marta Lacort-Beltrán, Adrián Alejandre, Sara Guillén, Marina Vilella, Xian Pan, Victoria Pueyo, Marta Ortin, Eduardo Esteban-Ibañez

**Affiliations:** 1 DIVE Medical S.L., 50009 Zaragoza, Spain; 2 Aragón Health Research Institute (IIS Aragón), 50009 Zaragoza, Spain; vicpueyo@gmail.com; 3 Department of Microbiology, Pediatrics, Radiology, and Public Health, Faculty of Medicine, University of Zaragoza, 50009 Zaragoza, Spain; 4 Ophthalmology Department, Miguel Servet University Hospital, 50009 Zaragoza, Spain

**Keywords:** eye tracking, performance, ophthalmic lenses, infrared filters, oculomotor control

## Abstract

Eye tracking (ET) technology is increasingly used in both research and clinical practice, but its accuracy may be compromised by the presence of ophthalmic lenses. This study systematically evaluated the influence of different optical prescriptions and lens treatments on ET performance using DIVE (Device for an Integral Visual Examination). Fourteen healthy participants underwent oculomotor control tests under thirteen optical conditions: six with varying dioptric powers and six with optical filters, compared against a no-lens control. Key parameters analysed included angle error, fixation stability (bivariate contour ellipse area, BCEA), saccadic accuracy, number of data gaps, and proportion of valid frames. High-powered spherical lenses (+6.00 D and −6.00 D) significantly increased gaze angle error, and the negative lens also increased data gaps, while cylindrical lenses had a moderate effect. Among filters, the Natural IR coating caused the greatest deterioration in ET performance, reducing valid samples and increasing the number of gaps with data loss, likely due to interference with the infrared-based detection system. The lens with basic anti-reflective treatment (SV Org 1.5 AR) also showed some deterioration in interaction with the ET. Other filters showed minimal or no significant impact. These findings demonstrate that both high-powered prescriptions and certain lens treatments can compromise ET data quality, highlighting the importance of accounting for optical conditions in experimental design and clinical applications.

## 1. Introduction

Eye tracking (ET) technology is transforming many areas of society, from research approaches (including data analysis, sports science, cognitive psychology, and reading studies) to clinical practice [1]. It enables objective, real-time assessment of visual responses with a level of accuracy unattainable by the human eye [2].

In clinical practice, the use of digital devices to assess visual function and diagnose impairments is becoming more and more common due to the lack of adequate tests for certain patient groups. This occurs especially in young children who are not able to understand or collaborate in order to obtain an accurate result with traditional diagnostic tools.

In this study, we focus on ET technologies that use infrared to record reflections from the cornea, pupil, or both [3]. In this way, they can determine the patient’s eye position, movements, and fixations to capture gaze information. This is especially useful when dealing with uncooperative patients, preverbal children, or children with disabilities who are unable to provide verbal or motor responses [4,5].

To overcome the limitations of conventional tests, several ET systems have been developed, such as Irisbond or Tobii, which are integrated into health solutions such as Gazelab, Bitbrain, or DIVE, whose development is based on ET technology and artificial intelligence.

To obtain reliable metrics from ET assessments, accuracy must be ensured through proper calibration for each participant. There are several reasons why accuracy may drop: the quality of the ET camera, miscalculation of the position of the eyes, participants’ characteristics (such as eye colour), or visual aids, among others [2,6]. The use of glasses with high prescriptions or with different types of coatings (or even with dirty surfaces) can interfere with this perception, leading to lower calibration quality [7] due to the reflections that occur on the lens’ surface [8].

In addition, any intermediate optical surface may introduce variability in the apparent position of the cornea. This unpredictability complicates the calibration process and, consequently, reduces the precision of measurements. Moreover, many ophthalmic lenses incorporate infrared light filters that are likely to interfere with the functioning of eye trackers. For this reason, the use of progressive lenses or certain anti-glare coatings that alter corneal reflections is commonly considered an exclusion criterion in ET studies, given the difficulty of reliably detecting the cornea [9]. However, to the best of our knowledge, no systematic study has evaluated and compared the effect of different lens types and treatments on ET.

The aim of this study is to determine whether the use of glasses with different prescriptions and optical filters affects the eye tracker’s ability to detect ocular position and, consequently, the accuracy of its metrics. To this end, participants will be evaluated both with and without different types of lenses using an ET device in order to assess how various lens types and filters may influence the system’s performance.

## 2. Materials and Methods

### 2.1. Participants

All participants included in this study were recruited from our Ophthalmology and Clinical Research Unit (Miguel Servet University Hospital, Zaragoza, Spain). All of them had to sign a written informed consent to be included in the study. All procedures during the study adhered to the tenets of the Declaration of Helsinki and were approved by the local ethics committee CEICA (Comité de Ética de la Investigación de la Comunidad Autónoma de Aragón).

Adults and teenagers between 15 and 45 years of age were eligible. None of them presented any recent ocular surgery or ocular disease. The maximum refractive error acceptable for the study was −1.00 D of myopia, +1.00 D of hyperopia, or 0.50 D of astigmatism, since no correction other than the examined lenses was used in the protocol. The minimum near-vision visual acuity with the LEA near-vision test at 65 cm was 0.8 decimal in the right eye. Any refractive error greater than those mentioned above, or lower near-vision VA were considered exclusion criteria. It was essential that every participant in the study was able to understand and follow the testing procedure.

### 2.2. Ophthalmic Lenses

The impact of 13 different optical conditions (1 control, 6 with different powers and 6 with different treatments) was evaluated on five key ET performance parameters. Each optical condition was applied under identical experimental conditions. The results were analysed comparatively using condition 0 (control, no lens) as a reference.

All lenses used in the study were sourced from INDO, and all information regarding their characteristics was obtained from their technical specifications [10].

#### 2.2.1. Power

Lenses with spherical and cylindrical powers are all made out of the same material. All lenses specified in Table 1 were analysed:

#### 2.2.2. Filter

Lenses with the filters specified in Table 2 were analysed.

### 2.3. DIVE

**Figure 1:**
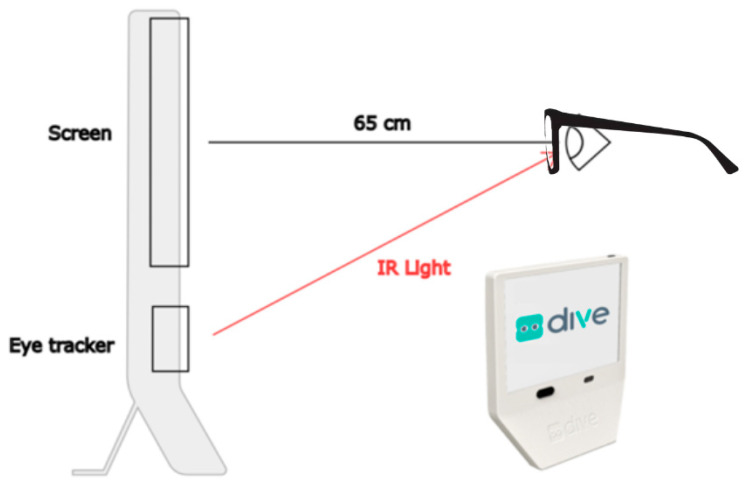
DIVE diagram showing a side view of the device, the position of the patients’ eyes at 65 cm from the screen, and the infrared light path from the eye tracker to the eyes.

Eye tracker performance was evaluated by performing an assessment of the oculomotor control (OMC) using DIVE (Figure 1). DIVE is a digital device that performs an automatic and comprehensive examination of the visual function. It has a 12-inch high-resolution touchscreen for visual stimuli display, corresponding to a visual angle of 28.46 degrees horizontally and 19.19 degrees vertically at a viewing distance of 65 cm (at which the tests are carried out) [11].

The device screen was calibrated weekly with the Spider X Pro calibrator (Datacolor, Rotkreuz, Switzerland). D65 illuminant was selected, ensuring that the white point luminance of the screen was set at 120 candelas per square metre (cd/m^2^). Gamma value (luminance intensity versus signal voltage) was controlled and established at 2.20, as recommended by Aslam [12].

The device performs an exam of several visual functions, such as oculomotor control, visual acuity, contrast sensitivity, and colour perception, without requiring verbal or motor feedback from the patients. It can, therefore, be performed with patients from 6 months of age, as well as children and adults with motor or cognitive issues.

The eye movements were collected by an ET to capture the patient’s response to those stimuli, with a temporal resolution of 120 Hz (120 samples per second). The ET used was a Tobii model 5L (Tobii, Stockholm, Sweden), which is a display-mounted eye tracker with IR and visible light eye detection that records both eyes and has no physical head movement restrictions (such as a chin rest). It operates with 850 nm infrared light. The firmware version of the ET was v1.36.3, and the DIVE software version was v1.1.47. As reported by Tobii, the eye tracker latency is <14 ms, the precision of the gaze position detection is <5 mm for 95% of the population, and the accuracy is <15 mm for 95% of the population.

The gaze data obtained from the eye tracker was processed by DIVE prior to its analysis, filling periods of missing gaze data shorter than 0.075 s using linear interpolation. Regarding data loss, we obtained a median of 6.6% of lost samples when no lens filter was used. In tests where a lens with a filter was applied, the median loss increased to 20%. Overall, the median loss across all tests was 9%.

### 2.4. Experimental Procedure

#### 2.4.1. Ophthalmological Assessment

Prior to the oculomotor control assessment with DIVE, an assessment of the patient’s refraction was carried out with an autorefractometer under mesopic conditions, as there were very restrictive refraction limits for participation in the study. A general ophthalmological assessment was also carried out to ensure good ocular health.

Once all the patient data had been collected and we had ensured that the patient met the inclusion criteria in terms of refraction, the near visual acuity test without optical correction was performed with the LEA chart at 65 cm under photopic conditions, as well as the ETDRS distance VA test.

#### 2.4.2. DIVE Exploration

In the room where the tests were carried out, there was no light source, only the illumination from the device screen.

The patient sat in front of the device with their eyes at 65 cm from the screen, a distance controlled by the device and maintained throughout the test.

Before starting the test, the examiner explained the entire procedure to the patient. During the test, the patient received no instructions other than those given by the device itself.

**Figure 2:**
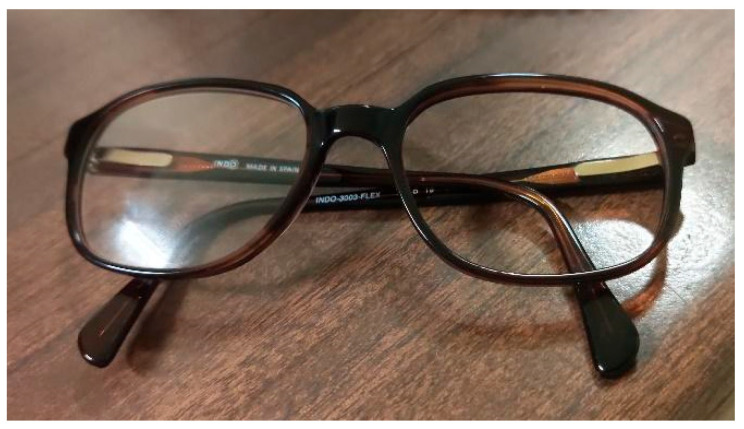
Frame with right eye lens. The left eye was covered with an external occluder during testing.

The DIVE evaluation was carried out with a frame in which the different prescription lenses and filters were mounted. A frame with interchangeable single-vision lenses was worn over the right eye (Figure 2). The left eye remained open but covered throughout the test in order to keep the extraocular muscles relaxed and prevent accommodation. A new calibration was performed for each lens before conducting the oculomotor control test.

The test consisted of performing the same oculomotor control test 13 times: 12 times with the different types of lenses placed on the right eye of the frame and one last time without any lens, with the frame still being used in this last condition to ensure consistency and to rule out any influence of the frame itself on the results. The order of the tests was randomised for each patient so that the results could not be influenced by test fatigue or learning.

Once the tests were completed, the results obtained were compared to those obtained from the same eye without any lens (baseline) in order to evaluate the influence of the lenses on the DIVE ET performance.

##### Calibration

The eye tracker provides the horizontal and vertical position of the gaze in pixels on the screen, where (0,0) corresponds to the top left corner of the screen. The ET must be calibrated before its use. In our case, we performed a nine-point binocular calibration in a dark room. The device displayed a 1-degree stimulus in nine positions evenly distributed across the screen. The stimulus was accompanied by sounds to facilitate the task. Each individual point was repeated, if necessary, until the ET reported a reliable calibration (with a limited number of repetitions to avoid an extensive calibration time).

**Figure 3:**
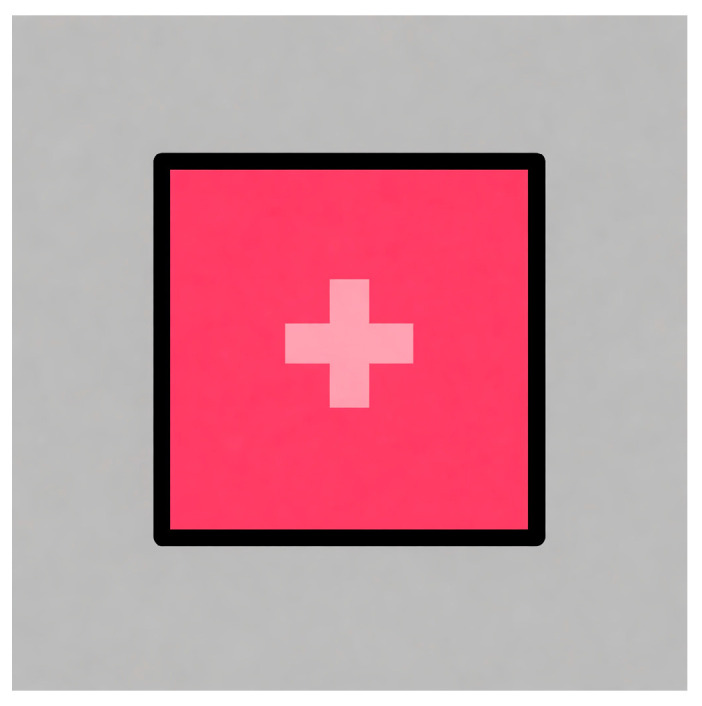
Square stimulus used to validate calibration.

After the calibration procedure, a validation test with 9 square figures measuring 2 degrees per side, with a cross in the middle to encourage the gaze to focus on a specific point in the centre (Figure 3), was performed. This was used to quantify the eye tracker’s exact accuracy and precision for each subject. Once these two steps had been performed, the calibration was finished, and the test was ready to start.

##### Oculomotor Control Tests

**Figure 4:**
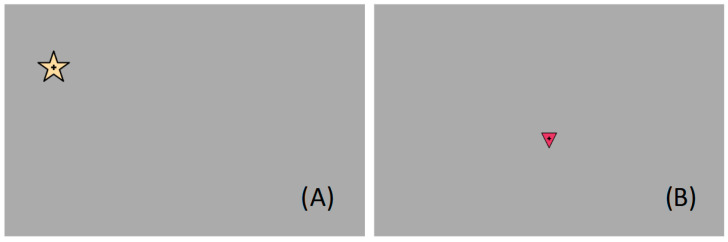
Image of the screen displayed during the fixation and saccades task (**A**) and smooth pursuit tasks (**B**).

The oculomotor control test was divided into fixation, saccades, and smooth pursuit tasks (Figure 4), all of which have been previously validated in earlier studies [11,13]. The fixation task consisted of an evaluation of long and short fixations. In the case of long fixations, a central stimulus was displayed in the middle of the screen for 12 s. The stimulus consisted of an interactive figure that spoke to the user, explaining that they should look at the screen and informing them of the next stimulus that will appear to help the participant maintain their attention during this long period of time.

For short fixations, a yellow star (Figure 4A) or square stimulus appeared at different positions on the screen for 3 s at a time (with no overlap). Saccades were measured during this time as well, using each stimulus as the starting point of the saccadic movement to the next.

Smooth pursuit was tested by following a stimulus (triangle, Figure 4B) that moved in a straight line on the screen. Movements were tested in the four main directions: from top to bottom and vice versa, and left to right and vice versa. The stimulus did not start moving until the ET detected that the patient was fixating on it.

#### 2.4.3. Statistical Analysis of the Data

The results collected to quantify the influence of the different lenses included five key parameters related to gaze accuracy and stability: angle error, fixation stability (bivariate contour ellipse area, BCEA), saccadic accuracy, number of gaps, and valid frames. Angle error was defined as the mean distance, in visual degrees, between the recorded gaze position and the validation stimulus. Fixation stability was assessed using the BCEA, which represents the area occupied by the gaze during stimulus presentation. BCEA was calculated as an ellipse containing 68% of the recorded samples. We thus included data not only from fixations but also between fixations by analysing data within a centred window during the long and short fixation tasks (11 s for the long fixation task; 2 s for the short task, as we eliminated the first 0.5 s and the last 0.5 s). Saccadic accuracy measures the correspondence between the performed and expected direction and amplitude of eye movements, so a score of 1 on this metric would correspond to perfect eye movement relative to the ideal. The number of gaps and the valid frames were directly computed from the raw data obtained from the eye tracker. The number of gaps was computed as the number of time periods during which the ET could not return the participant’s gaze position. No time restriction was applied to define a period of lost gaze as a data gap. Even a single lost frame between two frames with valid gaze information was considered a data gap. Finally, the proportion of valid frames (from 0 to 1) was evaluated. While the number of data gaps provides an integer value representing how many times the eye tracker lost track of the participant’s eyes, the proportion of valid frames indicates the fraction of the test duration during which the eye tracker successfully recorded the participant’s gaze position. The number of gaps and proportion of valid frames were computed using the ET data from the full exploration (the calibration validation, the short and long fixations, and the smooth pursuit tasks).

These last two metrics are not strictly correlated, as a single data gap may contain a large number of lost frames that drastically reduce the proportion of valid frames. Conversely, a large number of short data gaps may together account for only a small fraction of the total test duration. A high and correlated number of gaps and proportion of valid frames would indicate that the ET is experiencing difficulties throughout the entire scan, while an uncorrelated number, with few gaps and many valid frames, would indicate more of a one-off problem or a lack of attention on the part of the participant (or turning their head).

The five metrics analysed capture complementary aspects of ET performance. Angle error and fixation stability assess the system’s static accuracy and precision; saccadic accuracy reflects dynamic tracking performance; and the number of gaps and valid frames provides indicators of overall data quality and recording reliability. Given the homogeneous visual profile of participants and the identical testing conditions, these metrics can be interpreted as valid measures of the ET system’s performance rather than subject-dependent variability.

The results obtained for each of these parameters under the different optical conditions evaluated are presented below.

Statistical analysis was performed using IBM SPSS Statistics Version 25 (IBM Corp., Armonk, NY, USA) and R 4.5.1 (R Foundation for Statistical Computing, Vienna, Austria). The Shapiro–Wilk normality test was used to verify that the study variables had a normal distribution. Due to the different behaviour of the variables with respect to normality and a relatively small sample size, non-parametric statistics were used to detect differences between study variables for the different lenses used. As the same sample performed the same measurements using different filters and lenses (related or dependent sample), the Friedman test was used to detect whether there were differences between the control (without lens; 0) and all the lenses of different power and those with different filters, independently. If there were significant differences, a post hoc analysis (Wilcoxon signed-rank test) was performed to determine whether there were significant differences between the control and each of the optical power or filter lenses in order to identify which of them showed such a difference. The precision of the study estimates was assessed by calculating the maximum expected error for the most relevant quantitative variables, using the standard deviation observed in the sample and assuming a two-sided α error of 0.05 and a β error of 0.20. For each outcome, the precision was computed. This approach allowed us to quantify the expected accuracy of the estimates obtained with the final sample size and to compare it with the magnitudes of the differences found between the different lenses. Throughout the entire analysis, a significance level of *p*-values < 0.05 was applied to define statistical significance.

## 3. Results

A total of 14 patients were included in the study. The mean age was 26.86 years (the youngest was 18 years old and the oldest 40 years old) and the mean refractive error was 0.39 D in the spherical component and 0.27 D in the cylindrical component. None of them had visual pathologies, and all achieved a decimal visual acuity of 1.0 for both distance and near vision.

**Figure 5:**
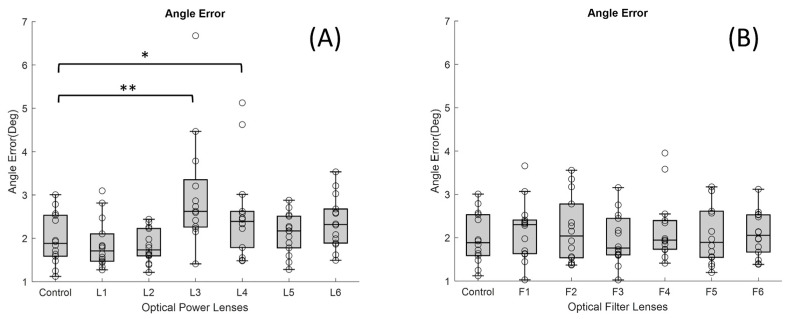
Boxplots showing the distribution of angle error values for each lens condition: (**A**) lenses with different dioptric powers and (**B**) lenses with different optical filters (* *p*-value < 0.05 and ** <0.001). In each boxplot, the black line represents the median, and the boxes indicate the interquartile range (IQR).

Regarding angle error, Figure 5A shows the angle error results for different powers and the control group, while Figure 5B shows the results for treatments. A significant increase in median angle error was observed in conditions with high dioptric power lenses, both positive and negative. L3 (+6 D) and L4 (−6 D) lenses showed the largest errors (*p*-value < 0.05 and <0.001, respectively); the median value for the control lens was 1.94 Deg (IQR 0.94), while L3 presented 2.60 Deg (IQR 1.09) and L4 2.39 Deg (IQR 0.83). Lenses with cylindrical components (L5 and L6) also showed increases (median values of 2.17 and 2.32 Deg, respectively), although the increases were more moderate. As for ophthalmic lenses with filters (F1–F6), minor variations were detected, and the differences found were not significant.

**Figure 6:**
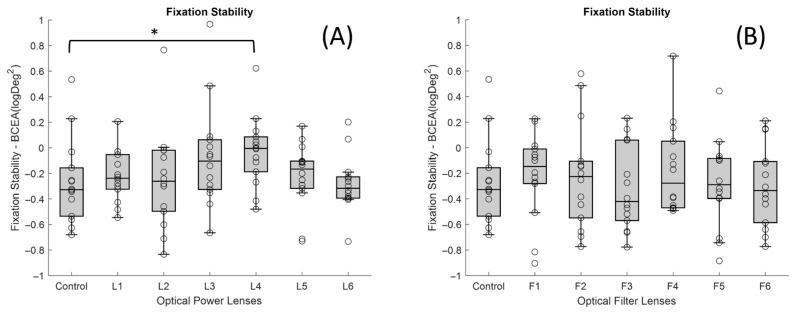
Boxplots showing the distribution of BCEA values for each lens condition: (**A**) lenses with different dioptric powers and (**B**) lenses with different optical filters (* *p*-value < 0.05). In each boxplot, the black line represents the median, and the boxes indicate the interquartile range (IQR).

The fixation stability increased in most of the powered lenses (Figure 6A), although the only one that showed a significant difference (*p*-value < 0.05) with respect to the control lens (median of −0.33 logDeg^2^ and IQR 0.38) was the L4 (median of −4.83 × 10^−3^ logDeg^2^ and IQR 0.27). This effect suggests greater fixation instability with that lens. In contrast, ophthalmic lenses with filters (Figure 6B) maintained fixation areas similar to the control condition, indicating less gaze dispersion.

**Figure 7:**
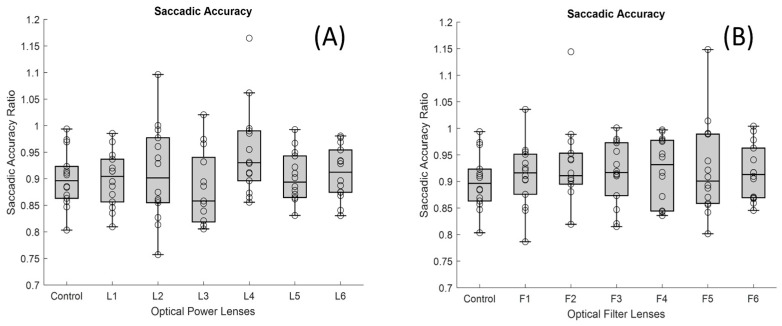
Boxplots showing the distribution of saccadic accuracy values for each lens condition: (**A**) lenses with different dioptric powers and (**B**) lenses with different optical filters. In each boxplot, the black line represents the median, and the boxes indicate the interquartile range (IQR).

With respect to the accuracy of saccadic movements, no significant differences were found for any of the lenses studied (Figure 7A,B).

**Figure 8:**
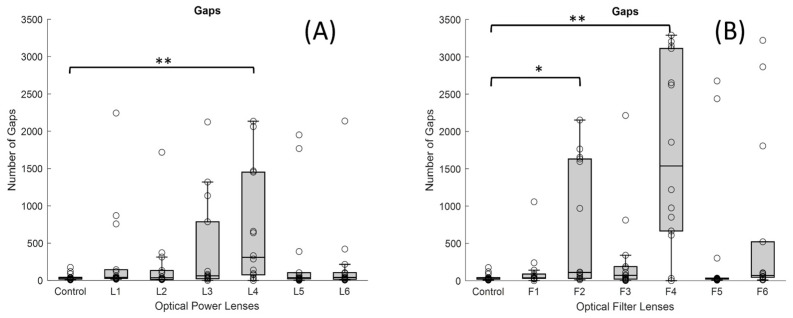
Boxplots showing the distribution of the number of gaps for each lens condition: (**A**) lenses with different dioptric powers and (**B**) lenses with different optical filters (* *p*-value < 0.05 and ** <0.001). In each boxplot, the black line represents the median, and the boxes indicate the interquartile range (IQR).

The greatest impact of the filters was observed in the number of gaps without samples received by the ET (Figure 8B). In particular, the F4 showed the highest number of gaps (median value of 1538 gaps and IQR 2446) compared to the control lens (*p*-value < 0.001), which showed practically no gaps in the recording. This filter prevents the wavelengths corresponding to the infrared from passing through, and thus, the infrared light from the ET was subject to interference in the transmission. F2 also showed an increased number of gaps. Its median value was 110.5 gaps, and IQR 1600, with a *p*-value of <0.05 in comparison with the control lens.

On the other hand, the number of gaps increased markedly in the presence of the high-powered negative lens, as shown in Figure 8A, the L4 lens (median value of 310 gaps and IQR 1376) compared to the control lens (median of 35.5 gaps and IQR 23), as the *p*-value was <0.001. However, in the high-powered positive lens, no significant differences were observed compared to the control lens.

**Figure 9:**
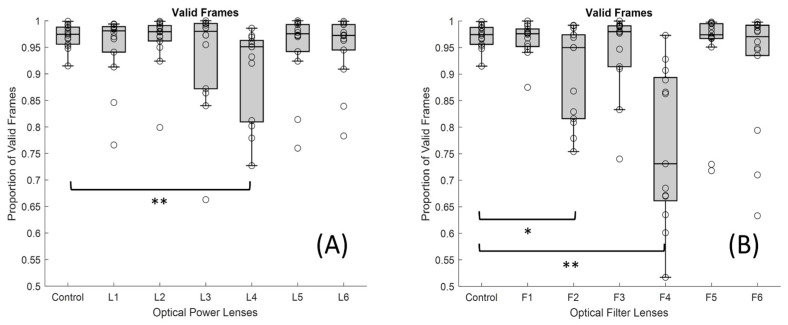
Boxplots showing the distribution of valid frame proportion for each lens condition: (**A**) lenses with different dioptric powers and (**B**) lenses with different optical filters (* *p*-value < 0.05 and ** <0.001). In each boxplot, the black line represents the median, and the boxes indicate the interquartile range (IQR).

Finally, with regard to the proportion of valid frames, it can be said that the only powered lens that showed significant differences compared with the control lens was the L4, with a median value of 0.951 and IQR of 0.154 (Figure 9A). As for the lenses with filters (Figure 9B), both the F2 and the F4 lenses showed a lower proportion of valid frames (median of 0.950 and IQR of 0.158 for the F2 and median of 0.731 and IQR of 0.232 for the F4) compared to the control situation (median of 0.975 and IQR of 0.02), as the *p*-value was <0.05 for F2 and <0.001 for F4. This indicates that there was a malfunction of the ET with the indicated lenses (L4, F2, and F4). It can be seen that all these gaps are distributed throughout the entire scan time; they do not occur at a specific moment. This clearly demonstrates that the increase in gaps and reduction in valid frames are caused by the lenses.

The sample size included in this study provides adequate statistical precision for the two primary quantitative variables analysed. For gaps, using a standard deviation of 47 units and assuming a two-sided α error of 0.05 and a β error of 0.20, the maximum estimation error was 35.2 gaps. This error margin is markedly smaller than the mean differences observed between the control group and the two lenses influencing the performance of the eye tracking the most (623 gaps with L4 and 1683 gaps with F4), indicating that the study is sufficiently powered to detect differences in clinically relevant magnitude. Similarly, for valid frames, the standard deviation was 0.022, and the calculated precision under the same α and β parameters was 0.0165. This level of error is negligible when compared with the observed between-group differences (0.07 between control lens and L4 and 0.21 between control lens and F4), again supporting that the sample size is more than adequate to ensure robust and reliable estimations. Taken together, these calculations confirm that the sample size is sufficient to sustain the validity of the statistical comparisons made.

## 4. Discussion

In this article, we demonstrate and quantify for the first time the effect of optical lenses and different filters on the functioning of the ET.

Different ophthalmic lenses—six with varying dioptric powers and six containing optical filters—were assessed for their impact on the performance of an ET device. Using the oculomotor control test in DIVE, we compared the metrics obtained with each lens to those from the same patient without lenses, under identical conditions.

The results show that the optical characteristics of the lenses significantly influence both the quality and accuracy of ET measurements. The high-powered spherical negative L4 lens (−6.00 D) was associated with poorer performance in variables such as angle error and number of gaps, fixation stability, and valid frames, while the high-powered spherical positive L3 lens (+6.00 D) showed worse results just for angle error.

We observed that the negative lens offers poorer quality in interaction with the ET compared to the positive lens, which could be associated with the difference in subjective pupil size caused by the power of the lens. In the case of the negative lens, the subjective pupil size is much smaller, which could make it difficult for the ET to collect valid samples due to the limited spatial resolution available to detect very small pupil images through the lens.

On the other hand, although the distance between the subject and the device remained constant at 65 cm, the placement of positive and negative lenses may affect the subjective distance recorded by the ET. The smaller pupil image produced by negative lenses could behave similarly to being physically farther from the device, and it is known that ET performance deteriorates at distances greater than the optimal working range [14].

The degradation in performance with high-power lenses can be attributed to several reasons. Firstly, it should be noted that any lens with power induces optical aberrations that produce distortion in the image. The higher the power of the lens, the greater these aberrations will be. Geometrical aberrations and thermal nonlinearities in high-power laser beams passing through lenses can impact beam quality [15].

Images seen through eyeglasses at different gaze directions are mainly influenced by three optical phenomena: image displacement, blur, and magnification [16]. Image displacement, or prismatic error in optometry, arises from changes in the line of sight, while blur occurs when the image of a viewing point is not punctual. Magnification, defined as the ratio between object and image height [16], becomes more pronounced in high-power lenses, where increased lens thickness enlarges the eye-to-lens separation. These factors affect the optical eye–lens–ET system and ultimately influence the quality of the ET samples collected from eye movements.

Ophthalmic lenses also influence pupil size, a factor that directly impacts the accuracy of ET. Variations in pupil size can lead to shifts in reported gaze position exceeding 2 degrees in camera-based eye trackers [17]. Moreover, the three aforementioned optical phenomena likewise affect both pupil size and the apparent target eccentricity, effects that become especially relevant with higher-power lenses and larger visual field eccentricities [18].

Although not analysed in this study, Concepción-Grande et al. [19] reported that the type of multifocal lenses may also influence the results. They evaluated the influence of different progressive lenses on visual quality by measuring visual acuity and using an ET-based system to analyse fixations, since ET provides objective and quantitative data that complements traditional VA assessment.

It can be concluded that, on the one hand, lenses with high dioptric power, both positive and negative (L3: +6.00 D and L4: −6.00 D), generate interference in the transmission of information from the ET to the eye and back. On the other hand, when it comes to filtered lenses, it should be noted that the Natural IR filter (F4) and the SV Org. 1.5 AR (F2) had the greatest impact on the quality of the samples collected by the ET. These filters demonstrated poorer performance in the number of gaps and valid frames. When it comes to the Natural IR filter, this effect can be attributed to direct interference between the filter applied in this lens and the principle of operation of ET systems based on near-infrared light. However, with reference to the SV Org. 1.5 lens, the ET’s performance could be related to the quality and spectral range of its multilayer AR coating. Simpler, low-cost AR coatings usually consist of only a few layers optimised for the visible spectrum, whereas premium AR coatings include multiple layers designed to extend their low-reflectance performance into the near-infrared range and to reduce angular dependence. As a result, the basic AR may increase reflectance or generate parasitic reflections in the infrared band, thereby compromising the eye tracker’s ability to reliably detect the pupil and corneal reflections [20].

This explanation is consistent with the data obtained, as the F2 condition showed a moderate but noticeable increase in gaps compared to both the control and other AR-coated lenses. Given that all experimental parameters remained constant, these differences can be reasonably attributed to the coating’s spectral inefficiency in the near-infrared range, rather than to procedural or setup factors.

The ET used for the study was a Tobii 5L model that uses emitters and cameras operating in the 850 nm wavelength to detect specific pupil and corneal reflections. The IR filter incorporated in these lenses is designed to attenuate or modify the transmission of this spectral range for eye protection or visual comfort purposes. This filtering affects both the light incident on the eye and the light returning to the system’s sensors, and it may compromise the detection of pupil or corneal reflections necessary for correct gaze point estimation.

Interestingly, although the infrared spectral transmission curves of INDO’s Natural IR and Energy Blue IR filters are practically identical, only the Energy Blue IR model allows the ET system to function correctly. This observation indicates that the problem is not solely related to the percentage of infrared transmission but rather to more complex optical factors that are not captured by standard spectral measurements. For instance, subtle differences in the coatings applied to the lens surface, such as the effectiveness of anti-reflective treatments, the presence of residual reflections, or the uniformity of coating thickness, may alter how infrared light is reflected or refracted as it enters and exits the lens. Additionally, the angular dependence of transmission, which describes how optical behaviour changes with the incidence angle of light, could significantly affect the infrared signals detected by the ET system, especially since eye trackers rely on precise corneal reflections. These nuances highlight that two lenses with nearly identical spectral transmission curves can still interact very differently with infrared-based technologies, emphasising the need for more detailed optical characterisation beyond standard transmittance data.

The multilayer coatings of this type of lens can induce internal reflection phenomena or optical interference, which further hinders eye segmentation by the ET software. Together, these factors might explain the observed deterioration in data quality when using this IR-filtered lens, highlighting the need to consider the spectral properties of optical treatments when designing studies based on ET technologies.

An exhaustive review of the scientific literature has been carried out in order to identify studies that evaluate the performance of ET systems in the presence of different types of ophthalmic lenses, both in terms of their dioptric powers and the optical treatments applied, including filters and coatings. However, no studies have been found that specifically and systematically address the technical limitations of these devices when used in combination with spectacles. This lack of studies highlights the relevance of the present work, which constitutes a first rigorous approach to the analysis of the impact that different types of lenses can have on the reliability and accuracy of the records obtained by eye trackers.

The value of this study lies not only in identifying which lenses are most likely to induce errors or loss of information in the capture of data with ET but also in demonstrating that the quantitative results provided by these devices when assessing certain visual functions may not be entirely accurate in specific optical conditions.

Data loss may make it more difficult to obtain reliable estimates of fixation stability or saccadic accuracy for each stimulus presented during the test. Because the final outcome for each metric is calculated as the median across stimuli, a reduced number of valid samples could increase the variance of the results. However, data loss by itself would not artificially increase fixation stability, as having fewer samples typically results in a smaller measured fixation area rather than a larger one. In our study, no significant differences were found in saccadic accuracy for any of the lenses examined (Figure 7).

This opens up a line of research of great interest and potential, allowing progress towards a more precise and critical interpretation of the data collected with ET technologies, especially in clinical and applied research contexts where visual precision is essential.

The main limitations of this study are as follows. First, the study had a relatively small sample size, which was a direct consequence of applying strict exclusion criteria to minimise potential biases, as well as the inherent difficulty of an exploratory protocol requiring each participant to complete 13 different lens and filter conditions. Second, each lens type was evaluated in isolation rather than in a more natural situation, where patients wear their own spectacles to correct refractive errors at the standard testing distance, allowing adaptation effects to be properly considered. Third, multifocal lenses were not included in the analysis, despite their widespread clinical use and potential impact on ET performance. Fourth, there was a lack of transmission profiles for the two filters analysed, which would be essential to gain an in-depth understanding of their differential behaviour, as both are designed to filter IR light. This aspect represents a limitation of the study and will be addressed in future research. Finally, lenses that combine both refractive power and optical filters, as typically found in real-world conditions, were not examined together but separately, limiting the generalisability of the findings. Future studies could also explore validation stimuli specifically adapted to each visual task (fixation, saccades, or smooth pursuit) to further reduce potential variability related to stimulus geometry and optimise ET performance assessment.

Despite these constraints, the results emphasise the importance of accounting for both the optical properties of lenses and their treatments when interpreting ET data. Such considerations are crucial to ensure the validity of results and to avoid misinterpretations that could compromise scientific evidence or clinical decision-making.

## 5. Conclusions

The main contribution of this study is to provide empirical evidence that the optical prescription required by each individual can influence the performance of ET systems, irrespective of their application (research, clinical, or data analysis).

It can therefore be concluded that certain lenses will affect the performance of the eye tracker. This study observed that particularly high-powered lenses (+6.00 D to −6.00 D) affect performance in terms of angle error. In addition, high-powered negative lenses also influence the correct execution of the ET with respect to the number of gaps, fixation stability, and valid frames. As far as filter lenses are concerned, the filter that showed the greatest interference with the ET was the Natural IR, which greatly deteriorated the data by increasing the gaps and decreasing the proportion of valid frames. The lens with basic anti-reflective treatment also showed some deterioration in interaction with the ET, mostly in terms of data gaps and valid frames.

## Figures and Tables

**Table 1:** Description of optical power for each type of lens.

Power Label	Power (D)
L1	+2.00 Sph OD
L2	−2.00 Sph OD
L3	+6.00 Sph OD
L4	−6.00 Sph OD
L5	+3.00 Cyl 180° OD
L6	−3.00 Cyl 90° OD

**Table 2:** Explanation of the treatments for each type of filter and their transmission profiles [10].

Filter Label	Filter	Treatment	Lens Transmission Profile
F1	Monofocal (SV) Org. 1.5	Basic uncoated lens	The lens transmission profile is not available in the INDO catalogue
F2	SV Org. 1.5 AR	Basic anti-reflective coating lens	The lens transmission profile is not available in the INDO catalogue
F3	Natural Super Clear	Anti-reflective filter with invisible effect. Reflects 0.15% of light. Maximum transmittance (99.6%). High stability. Angular control of surface reflection at all viewing angles	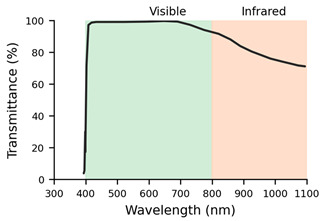
F4	Natural IR	Anti-reflective filter. Protects against all solar radiation, including infrared radiation. 60% blocking of infrared A radiation. Transparency, uniform green residual reflection.	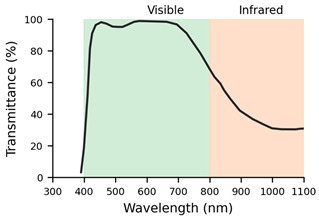
F5	Natural 10	Anti-reflective filter. Reduces reflections from the frame on the lens to improve aesthetics and visual comfort. Scratch resistance and slight green reflection.	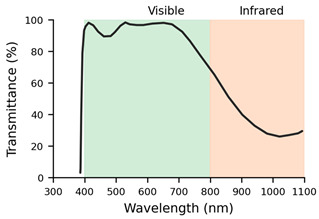
F6	Energy Blue	Blue light filter. Protects against excessive blue light from electronic devices and all solar radiation, including infrared.	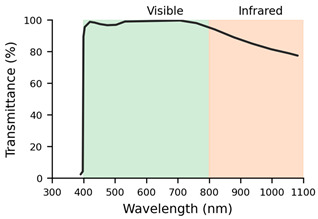

## Data Availability

The raw datasets generated and analysed during the current study are not publicly available at this time but may be obtained from the corresponding author on reasonable request.

## Abbreviations

The following abbreviations are used in this manuscript:

ET	Eye tracking
BCEA	Bivariate Contour Ellipse Area
DIVE	Device for an Integral Visual Examination
IQR	Interquartile range
OMC	Oculomotor Control
IR	Infrared
OD	Oculus Dexter
AR	Anti-reflective coating
SV	Single Vision
Org.	Organic
D	Diopters
Fig.	Figure
s	Seconds
